# Federated Learning with Assured Privacy and Reputation-Driven Incentives for Internet of Vehicles

**DOI:** 10.3390/s26051720

**Published:** 2026-03-09

**Authors:** Jiayong Chai, Mo Chen, Wei Zhang, Xiaojuan Wang, Jiaming Song

**Affiliations:** 1 School of Electronic Engineering, Beijing University of Posts and Telecommunications, Beijing 100876, China; 2 College of Computer Science, Beijing Information Science and Technology University, Beijing 100083, China; 20202459@bistu.edu.cn; 3 China Datang Corporation Science and Technology General Research Institute Ltd., Beijing 100040, China; zhangwei@cdt-kxjs.com; 4 School of Cyberspace Security, Beijing University of Posts and Telecommunications, Beijing 100876, China; wj2718@bupt.edu.cn; 5 State Key Lab Networking & Switching Technol, Beijing University of Posts and Telecommunications, Beijing 100876, China; songjm@bupt.edu.cn

**Keywords:** federated learning, blockchain, gradient privacy, incentive mechanism, zero-knowledge proof, Internet of Things

## Abstract

Cross-domain data collaboration is a core requirement for the intelligent development of critical areas such as the Internet of Vehicles and intelligent transportation systems. In this scenario, vehicles and various sensors deployed roadside continuously generate massive amounts of time-series data, yet this data often forms “data silos” due to privacy regulations and a lack of trust between collaborating entities. Existing integrated schemes combining “Federated Learning + Blockchain” have achieved a certain degree of process traceability and automated payments, but risks of gradient-level privacy leakage persist, and inflexible and delayed incentive mechanisms result in low participation quality. To systematically address these bottlenecks, this paper proposes the Federated Learning with Assured Privacy and Reputation-Driven Incentives (FLARE) architecture, whose core innovation lies in the native integration of cryptographic security and mechanism design theory. It includes the Secure and Faithfully Executed Gradient aggregation (SafeGrad) protocol, which integrates partial homomorphic encryption and zero-knowledge proofs to provide verifiable privacy guarantees for gradient contributions while enabling efficient secure aggregation, defending against inversion attacks at the source; alongside this, it includes the Economy-on-Chain incentive (EconChain) mechanism, which designs an on-chain economic system based on blockchain, achieving precise measurement and sustainable incentivization of training process contributions through fine-grained instant micro-rewards and a dynamic reputation model. Experiments show that, compared to baseline schemes, FLARE can effectively enhance node participation enthusiasm and contribution quality without compromising model accuracy, providing a new paradigm with both strong security and high vitality for the trusted and efficient circulation of data.

## 1. Introduction

With the rapid development of the Internet of Things, in areas like the Internet of Vehicles and intelligent transportation, the massive perceptual data generated by vehicles, roadside devices, and traffic control centers forms difficult-to-collaborate “data silos” due to dispersed ownership, strict privacy regulations, and a lack of trust, constraining the enhancement of global traffic situational awareness and collaborative decision-making capabilities [1,2].

To address the challenge of data collaboration and sharing while protecting data privacy, the federated learning framework [3] has been proposed. However, users’ private data may still be leaked through collusion attacks, model inversion attacks, etc., when local gradients are uploaded to the server for aggregation, making gradient protection particularly important [4]. Simultaneously, different users have varying data quality, computational resources, and communication capabilities, leading to different costs for local training, yet they receive the same global model. This weakens the participation willingness of high-quality rational users, subsequently affecting global model quality. Therefore, a suitable incentive mechanism is also indispensable [5,6,7,8].

As will be detailed in Section 2, existing approaches that combine privacy protection and incentives are often insufficient: they either risk gradient-level privacy leakage or rely on inflexible incentive mechanisms, resulting in low participation quality. To systematically overcome these limitations, this paper proposes the Federated Learning with Assured Privacy and Reputation-Driven Incentives (FLARE) architecture, whose core innovation lies in the native integration of cryptographic security and mechanism design theory. It includes the Secure and Faithfully Executed Gradient aggregation (SafeGrad) protocol, which integrates partial homomorphic encryption and zero-knowledge proofs to provide verifiable privacy guarantees for gradient contributions while enabling efficient secure aggregation, defending against inversion attacks at the source; alongside this, it includes the Economy-on-Chain incentive (EconChain) mechanism, which designs an on-chain economic system based on blockchain, achieving precise measurement and sustainable incentivization of training process contributions through fine-grained instant micro-rewards and a dynamic reputation model.

The remainder of this paper is organized as follows: Section 2 comprehensively reviews related work on privacy-preserving federated learning and incentive mechanisms. Section 3 provides an overview of the FLARE system architecture and workflow. Section 4 and Section 5 detail the SafeGrad protocol and EconChain mechanism, respectively. Section 6 presents experimental evaluation and analysis. Section 7 discusses the results, limitations, and future work. Finally, Section 8 concludes the paper.

## 2. Related Work

This section reviews the research on privacy protection technology and incentive mechanisms of the main “Federated Learning + Blockchain” solutions, identifies key limitations in current approaches, and clarifies the research gap that FLARE addresses.

### 2.1. Privacy-Preserving Techniques in Federated Learning

Homomorphic encryption (HE) technology is widely used in federated learning for privacy protection. Phong et al. [9] were among the first to use the Paillier encryption scheme to protect local gradients in federated learning, but this scheme requires online collaborative decryption by clients, incurring significant communication overhead. Ma et al. [10] proposed a more efficient batch Paillier encryption scheme to mitigate ciphertext expansion, but its decryption process still relies on a trusted center, creating a single point of trust. Li et al. [11] proposed a multi-key homomorphic encryption method, allowing clients more flexible participation, but the system complexity and communication costs are higher. Research by Wibawa et al. [12] and Wang et al. [13] further demonstrated the potential of HE in federated learning for privacy-sensitive scenarios like healthcare. However, these schemes fail to address how to verify client contributions while protecting privacy [14,15,16].

Alternative approaches such as Secure Multi-Party Computation (SMPC) and Differential Privacy (DP) have also been explored. SMPC enables secure aggregation through cryptographic protocols but often involves multiple communication rounds and high computational overhead. Efficient secure aggregation protocols [17,18] have been proposed to reduce client-side overhead, yet they do not address contribution verifiability. DP adds calibrated noise to gradients to provide statistical privacy guarantees, but at the expense of model accuracy and utility. A common limitation across these privacy-preserving techniques is their inability to provide verifiable proof of the correctness or quality of contributions without compromising privacy. Recent advances in verifiable federated learning [19,20,21] have explored cryptographic proofs of honest execution, but often at the cost of significant communication or computation overhead.

### 2.2. Incentive Mechanisms for Federated Learning

Blockchain’s trusted recording and automated smart contracts are often used to build incentive mechanisms in federated learning. Wu et al. [22] proposed a framework using blockchain to record the training process and distribute token rewards, but this incentive is based solely on final model accuracy, unable to provide immediate feedback on contribution during training. Wang [23] proposed contribution evaluation based on Shapley value for fairness, but its computational overhead is enormous and requires aggregating intermediate results with weak protection, failing to protect privacy. An et al. [24] proposed using blockchain consensus to select participants and aggregate models, but its privacy-preserving computations are overly complex, limiting practicality. Wang et al. [25] proposed a committee scoring-based incentive mechanism, but this mechanism cannot resist the risk of committee collusion. Recent reputation-based mechanisms [26,27] demonstrate the importance of robust reputation updates against whitewashing and sybil attacks. Li et al. [28] proposed an explainable and privacy-preserving FL model for threat detection in cyber–physical–social systems, integrating SHAP-based explainability with differential privacy. Existing schemes have shortcomings in both the timeliness and fairness of incentive mechanisms, and they fail to effectively protect privacy during contribution evaluation [29,30,31]. Game-theoretic models of FL incentives have been refined in [32,33,34], considering reputation dynamics, collusion, and long-term participation.

### 2.3. Research Gap and FLARE’s Positioning

The literature review reveals a persistent disconnect: privacy-preserving techniques (e.g., HE and DP) often sacrifice the ability to verify the authenticity and quality of contributions, while incentive mechanisms (e.g., blockchain-based rewards) often require access to plaintext gradients or intermediate results, compromising privacy during contribution assessment. This disconnect is particularly problematic for high-stakes, privacy-sensitive applications like the Internet of Vehicles, where both strong privacy guarantees and fair, timely incentives are essential for sustainable collaboration.

FLARE bridges this gap through its native co-design of the SafeGrad protocol and EconChain mechanism. Unlike prior works that treat privacy and incentives as separate modules, FLARE embeds verifiability into privacy protection by using zero-knowledge proofs (zk-SNARKs) to allow for contribution assessment on encrypted gradients without decryption and ensure that each participant’s update is provably meaningful. FLARE embeds privacy protection into incentive distribution by ensuring that reward calculations are based solely on verified, yet private, contributions. FLARE creates a closed-loop system where verified contributions directly feed into a dynamic reputation model and instant micro-rewards, fostering sustainable high-quality participation.

Thus, FLARE offers a novel paradigm that moves beyond the privacy-or-incentives trade-off, providing a unified solution for trusted and efficient data circulation in IoV and similar cross-domain environments.

## 3. FLARE System Overview

In the Internet of Vehicles, the perception data continuously generated by on-board units, roadside units, and traffic monitoring sensors is crucial for achieving collaborative perception. However, due to data privacy restrictions and a lack of mutual trust among device operators, this data is often isolated in “data silos” and cannot be used for data collaboration. To address this, this paper proposes FLARE, a federated learning system integrating cryptographic security and on-chain incentive mechanisms.

FLARE comprises four core roles:

- Data holder nodes: It can be a vehicle-mounted unit, a road test unit, responsible for local model training, running the secure aggregation protocol to encrypt gradients and generate proofs, and participating in the dynamic incentive and reputation system.
- Secure aggregation server: A semi-honest computing party that runs the aggregation phase of the secure aggregation protocol, performing aggregation operations on encrypted gradients, but unable to decrypt any single client’s gradient.
- Smart contract group: Deployed on the blockchain, coordinating the normal operation of the system, specifically including (1) Task Management Contract—manages the publication and lifecycle of federated learning tasks; (2) Secure Aggregation Verification Contract—verifies the gradient contribution proofs submitted by clients; and (3) Dynamic Incentive Core Contract—distributes incentives, manages reputation, and dynamically selects participating nodes.
- Task requester: The initiator of the task and the owner of the final model, it can be a traffic management center, a service provider, or an automobile manufacturer. The requester providing the initial model and incentive budget.

**Figure 1:**
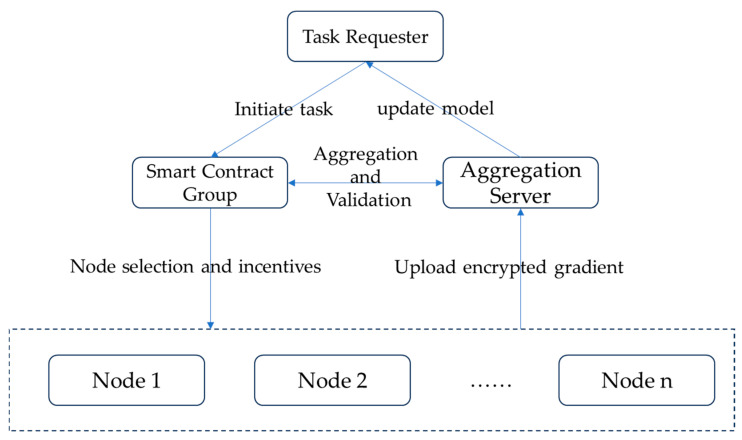
The FLARE system workflow: An integrated pipeline, from task initialization to incentive distribution.

The workflow of the FLARE system is shown in Figure 1, mainly comprising the following stages. (1) Initialization and registration: The task requester publishes a task. Data nodes register their identities with the blockchain and stake a deposit. (2) Committee selection: Before each training round begins, the Dynamic Incentive Core Contract dynamically selects the client committee for this round based on nodes’ reputation and resources. (3) Training and proof generation: Selected clients compute gradients using local data in this round. After computation, they generate encrypted gradients and corresponding contribution zero-knowledge proofs and then upload them to the aggregation server. (4) Secure aggregation and verification: The aggregation server performs homomorphic aggregation on the ciphertexts and submits the aggregation result to the blockchain. The Secure Aggregation Verification Contract verifies the validity of each client’s proof. (5) Model update and incentivization: After verification passes, the aggregation result is authorized for decryption and used to update the global model. Simultaneously, the Dynamic Incentive Core Contract distributes micro-rewards for this round based on the verified contribution proofs and updates the reputation values of all participating nodes. (6) Iteration and termination: Repeat steps 2–5 until the model converges or reaches the preset number of rounds. The task completes and final rewards are settled.

## 4. Gradient Secure Aggregation Protocol

Existing federated learning schemes implementing secure aggregation face issues of privacy leakage and unverifiable contributions. To this end, this paper proposes the SafeGrad (Secure and Faithfully Executed Gradient aggregation protocol) protocol, constructing a cryptographic framework that simultaneously ensures gradient confidentiality and provides verifiable contribution measurement by integrating zero-knowledge proofs and partial homomorphic encryption.

### 4.1. Problem Formalization and Threat Model

Consider a federated learning system consisting of N data-holding clients $\left\{C_{1},C_{2},\ldots ,C_{N}\right\}$; a semi-honest (Honest-but-Curious) central aggregation server, S; and a blockchain network, B, serving as a trusted coordination and verification layer. The system operates in synchronous rounds. In round t, a client committee, $C_{t}\subseteq \{C_{i}\}$, dynamically selected by smart contracts, participates in this round’s training.

Each client ($C_{i}$) holds a private local dataset, $D_{i}$. In round t, given the global model parameters, $w_{t}$, client $C_{i}$ computes its local stochastic gradient, $g_{i}^{t}=\nabla F_{i}(w_{t};\xi_{i})\in R^{d}$, via local stochastic gradient descent, where $\xi_{i}$ is a random batch sampled from $D_{i}$.

The design of this protocol needs to guarantee security under a threat model containing three types of adversaries. (1) Semi-honest central aggregation server, S: It will strictly follow the protocol specifications for computation and communication but will attempt to infer each client’s private gradient, $g_{i}^{t}$, or original data information from all received intermediate information (e.g., encrypted gradients and proofs). (2) Byzantine clients: At most, f malicious clients may collude and arbitrarily deviate from the protocol. However, the zero-knowledge proof only enforces a lower bound on the gradient norm; it does not verify the semantic correctness of the gradient direction. Thus, a client could potentially submit a gradient that satisfies the norm threshold but is directionally harmful. FLARE mitigates such attacks through two complementary mechanisms: (i) secure aggregation dilutes any individual malicious update, and (ii) the reputation system penalizes clients that consistently harm model performance. (3) Passive external eavesdroppers: These are capable of intercepting communication content on all network channels.

Quantifying Byzantine resilience: Let the committee size in round t be $K=|C_{t}|$, and suppose that up to b=f⋅K of the selected participants are malicious (0≤f<0.5). The secure aggregation protocol averages all submitted gradients, so the deviation introduced by malicious updates is bounded by $f\cdot {max}_{i}∥g_{i}^{t}∥$. FLARE mitigates such attacks through two complementary mechanisms:

1. Aggregation dilution: Even if malicious clients submit arbitrarily large or directionally harmful gradients, their influence is diluted by averaging with honest contributions. The effective perturbation after aggregation is at most $f\cdot G_{max}$, where $G_{max}={max}_{i}∥g_{i}^{t}∥$ is the maximum gradient norm in the round.
2. Reputation-based filtering: Clients that consistently cause model degradation (e.g., by submitting directionally harmful gradients) receive lower rewards, reducing their performance score ($R_{{\mathrm{pref}}_{i}}^{t}$) and, consequently, their reputation ($R_{{\mathrm{rep}}_{i}}^{t}$). Over time, such nodes are selected less frequently, further limiting their impact.

For convergence, consider a standard assumption in federated learning: the loss function, F(w), is L-smooth and μ-strongly convex. Under this assumption, if the deviation per round is bounded by ϵ, the optimality gap after T rounds satisfies the following:

(1) $$E[F(w_{T})-F(w^{*})]\leq \frac{L}{2}(1-\mu \eta {)}^{T}∥w_{0}-w^{*}{∥}^{2}+\frac{\eta \epsilon^{2}}{2\mu },$$

where η is the learning rate. The term $\frac{\eta \epsilon^{2}}{2\mu }$ represents the steady-state error due to Byzantine perturbations. To keep this error below a tolerable threshold, δ, we require $f\cdot G_{max}\leq \epsilon \leq \sqrt{\frac{2\mu \delta }{\eta }}$. Thus, the maximum tolerable fraction of malicious clients is as follows:

(2) $$f^{*}=\frac{1}{G_{max}}\sqrt{\frac{2\mu \delta }{\eta }}.$$

In our experiments, η=0.01, μ≈0.1 (estimated from ResNet-18 on CIFAR-10), and δ=0.01 (1% accuracy loss) yield $f^{*}\approx 0.14$, consistent with the empirical observation that f=0.1 causes a less than 1.5% accuracy drop. For larger committees, the bound improves because $G_{max}$ is averaged over more samples, reducing per-client influence. A rigorous extension to non-convex models is left for future work.

As an extension consideration, FLARE provides layered defenses against a malicious server: First, the server cannot decrypt individual client gradients alone; decryption requires collaboration from at least t out of n independent notaries (t-out-of-n threshold scheme). Second, client contribution proofs are verified by the Secure Aggregation Verification Contract on the blockchain, not by the server. The server cannot bypass this verification to accept invalid contributions. Third, the server must submit the aggregated ciphertext and its hash to the blockchain, creating a tamper-evident record. While verifiable aggregation proofs could further enhance accountability, this design significantly raises the cost of undetected malicious behavior. This extended consideration addresses practical deployment concerns where the aggregation service may be operated by a third-party cloud provider with potential conflicting interests.

### 4.2. Verifiable Contribution Measurement Based on Zero-Knowledge Proofs

In federated learning, fair and effective incentive mechanisms rely on accurate assessment of client contributions. This study selects the squared L2 norm of the gradient $g_{i}^{t}$, $S_{i}^{t}=∥g_{i}^{t}{∥}_{2}^{2}=\sum_{j=1}^{d}(g_{i,j}^{t}{)}^{2}$, as it is the core proxy variable for contribution measurement. This choice is justified by the convergence theory of stochastic gradient descent: under convex optimization assumptions, the expected loss function decrease is proportional to the squared gradient norm. However, directly requiring clients to upload $S_{i}^{t}$ or $g_{i}^{t}$ would directly leak privacy information and cannot verify its authenticity. Clients might falsely report a higher $S_{i}^{t}$ to obtain more rewards.

To resolve this contradiction, this study introduces zk-SNARK (Groth16). The core idea is that a client can generate an extremely concise and easily verifiable cryptographic proof, $\pi_{i}^{t}$, to prove to the verifier (smart contract) a mathematical statement, “I know a secret vector, $g_{i}^{t}$ ,such that $∥g_{i}^{t}{∥}_{2}^{2}\geq \theta$ holds,” without leaking any information about $g_{i}^{t}$ during the proof process.

#### 4.2.1. Arithmetic Circuit Construction

First, the statement to be proven needs to be transformed into an arithmetic circuit, C, processable by zk-SNARK. This study adopts the Quadratic Arithmetic Program (QAP) representation method. * Public Inputs (Statement): Contribution threshold θ (a public scalar). * Private inputs (witness): Gradient vector $g_{i}^{t}=(g_{1},g_{2},\ldots ,g_{d})$. * Circuit logic: (1) For j=1 to d, compute the square term $s_{j}=g_{j}*g_{j}$. (2) Compute the accumulated sum $S=\sum_{j=1}^{d}s_{j}$. (3) Introduce an auxiliary variable, diff=S−θ. 4. The circuit must prove diff=S−θ≥0. Direct inequality checks are not native to arithmetic circuits; therefore, a range proof is employed. Specifically, S and θ are interpreted as elements of a finite field, $F_{r}$, and the constraint, $diff\in [0,2^{L}-1]$, is enforced by decomposing diff into its binary representation within the circuit and verifying that each bit is indeed binary: $diff=\sum_{j=0}^{L-1}b_{j}\cdot 2^{j},\;b_{j}\in \{0,1\}.$ This ensures diff≥0 without restricting it to perfect squares. While the presentation here is schematic, an actual implementation uses efficient range-proof techniques to minimize the number of constraints. The choice of L must be large enough to cover the maximum possible value of S−θ, which is determined by the gradient bounds and the fixed-point scaling factor.

During the system initialization phase, a trusted setup runs the $\mathrm{Setup}(1^{\lambda },C)$ algorithm, generating a key pair: the proving key, pk; and the verification key, vk. The trusted setup can be executed as a one-time MPC ceremony where multiple parties contribute randomness. The setup remains secure as long as at least one participant is honest and destroys their secret contribution. For deployments where organizing an MPC ceremony is impractical, FLARE’s modular design allows for substitution with transparent zk-SNARK systems (e.g., PLONK and Bulletproofs) that eliminate the trusted setup entirely. This study assumes an MPC-based setup. vk will be deployed to the blockchain’s smart contract, while pk is made public to all clients.

A critical aspect of the implementation is the alignment between the plaintext space of the Paillier encryption ($Z_{n}$, where n is the RSA modulus) and the scalar field,$\;F_{r}$, of the Groth16 zk-SNARK (with prime r). Gradients are encoded as fixed-point numbers scaled by $2^{L}$, producing integer representations that must be interpreted correctly in both domains. To guarantee that no conflicting modular reductions occur, all encoded values must be strictly less than both n and r. This condition can be expressed as follows: $\forall i:\;2^{L}\cdot ∥g_{i}^{t}{∥}^{2}<min(n,r),$ because the squared norm, $S_{i}^{t}=∥g_{i}^{t}{∥}^{2}$, is the value that appears in the SNARK circuit, and the inequality, $S_{i}^{t}\geq \theta$, is verified there.

In the experimental setup of this work, the parameters are chosen as follows. Fixed-point scaling factor: L=16 (i.e., $2^{L}=65,536$). Gradient norm bound: For the models used (CNN, ResNet-18, and DenseNet-121), the observed squared gradient norms, $∥g_{i}^{t}{∥}^{2}$, rarely exceed $10^{3}$. Paillier modulus: n is a 2048-bit RSA modulus; thus, $n>2^{2048}$. SNARK curve: The BN254 curve is employed, whose scalar field modulus is $r\approx 2^{254}$. Consequently, $2^{L}\cdot ∥g_{i}^{t}{∥}^{2}\approx 6.5\times 10^{7}\approx 2^{26}\ll 2^{254}<min(n,r).$

Hence, the consistency condition is satisfied with a safety margin of more than $2^{228}$ orders of magnitude. For larger models where gradient norms may be larger, the same analysis can be used to adjust L or choose cryptographic parameters with larger moduli. Table 1 summarizes the relationship between typical model sizes, achievable gradient norms, and the required parameter choices to maintain consistency. A full exploration of the optimal trade-offs between precision, security, and performance is left for future work.

#### 4.2.2. Proof Generation and Verification

In round t, client $C_{i}$ performs the following steps. (1) Local computation: Compute local gradient, $g_{i}^{t}$, and its squared norm, $S_{i}^{t}=∥g_{i}^{t}{∥}_{2}^{2}$. (2) Proof generation: Invoke the $\mathrm{Prove}(pk,(g_{i}^{t},\theta ),⌀)$ algorithm. This algorithm takes the proving key, pk; public input, θ; and private witness, $g_{i}^{t}$, as inputs, and outputs a concise proof $\pi_{i}^{t}=(\pi_{A},\pi_{B},\pi_{C})\in G_{1}^{2}\times G_{2}$, where $G_{1}\;and\;G_{2}$ are elliptic curve groups. (3) Submission: The client submits the tuple $\left(S_{i}^{t},\pi_{i}^{t}\right)$ to the blockchain network.

Upon receiving the proof, the smart contract performs on-chain verification. (1) Verification: Invoke the $\mathrm{Verify}(vk,(\theta ,S_{i}^{t}),\pi_{i}^{t})$ algorithm. This algorithm, based on the bilinear pairing $e:G_{1}\times G_{2}\to G_{T}$, verifies whether the following equation holds:

(3) $$e(\pi_{A},\pi_{B})=e(\pi_{C},g_{2})\cdot e(vk_{\theta },g_{2}{)}^{S_{i}^{t}-\theta },$$

where $g_{2}$ is the generator of group $G_{2}$, and $vk_{\theta }$ is the part of the verification key, vk, corresponding to the public input, θ (generated during the trusted setup phase based on circuit, C). This equation verifies that proof $\pi_{i}^{t}$ is indeed a valid proof for circuit (C) with public inputs $\left(\theta ,S_{i}^{t}\right)$ and some private witness, $g_{i}^{t}$. (2) Result: If verification passes, the contract is convinced that client $C_{i}$ indeed possesses a legitimate gradient vector, $g_{i}^{t}$; the calculation of its squared norm, $S_{i}^{t}$, is correct; and it is no less than θ.

Equation (3) presents a schematic representation of the verification logic that is intended to convey the high-level idea that the proof, $\pi_{i}^{t}$, cryptographically binds the public input, $S_{i}^{t}$, to the private witness, $g_{i}^{t}$. In the actual implementation, the standard Groth16 verification equation is used: $e(A,B)=e(\alpha ,\beta )\cdot e\left(\sum_{i=0}^{l}a_{i}vk_{i},\;\gamma \right)\cdot e(C,\delta ),$ where (A,B,C) are the proof elements, α,β,γ,δ are the common reference string elements, $a_{0}=1$ and $a_{1},\ldots ,a_{l}$ are the public inputs (here, θ and $S_{i}^{t}$), and $vk_{i}$ represents the corresponding verification key components. The correctness of the verification follows from the properties of the underlying QAP.

Equation (3) is intentionally simplified to convey the core idea that the public input, $S_{i}^{t}$, is cryptographically bound to the proof. In the actual implementation, the verification key, vk, consists of multiple components, $vk_{0},vk_{1},\ldots ,vk_{l}$, and the public inputs $\left(\theta ,S_{i}^{t}\right)$ are combined as $\theta \cdot vk_{1}+S_{i}^{t}\cdot vk_{2}$ (plus constant term $vk_{0}$). The exact linear combination is generated during the trusted setup and embedded in the verification contract. Interested readers are referred to the Groth16 source code for the precise implementation details [35].

When a client honestly uses a $g_{i}^{t}$ satisfying $S_{i}^{t}\geq \theta$ to generate the proof, verification will certainly pass. That is, any algorithm capable of generating a valid proof, $\pi_{i}^{t}$, necessarily “knows” a corresponding private witness, $g_{i}^{t}$, such that $S_{i}^{t}=∥g_{i}^{t}{∥}_{2}^{2}$ holds. This cryptographically guarantees that the contribution value, $S_{i}^{t}$, is by no means fabricated, but originates from genuine local computation, while $\pi_{i}^{t}$ does not leak any information about $g_{i}^{t}$. The verifier only learns that the statement is true. This algorithm provides a foundation for contribution measurement for subsequent incentive mechanisms while protecting gradient privacy.

### 4.3. Secure Aggregation Protocol Based on Partial Homomorphic Encryption

The contribution proof ensures the verifiability of gradient “quality” (i.e., its value), while the aggregation process of gradient “value” (i.e., its numerical vector) still requires privacy protection. This paper employs the Paillier encryption scheme, which satisfies additive homomorphism, to build the secure aggregation protocol. In this phase, inflated gradients are averaged with contributions from other participants, limiting their distortive effect on the global model. This section first elaborates on the cryptographic theory underlying the protocol design, and then it presents the specific protocol steps.

#### 4.3.1. Protocol Theoretical Basis

This study employs the Paillier cryptosystem, a partial homomorphic encryption scheme satisfying additive homomorphism. For any two plaintexts, $m_{1},m_{2}\in Z_{n}$, the following property holds: $\mathrm{Enc}(pk,m_{1})\cdot \mathrm{Enc}(pk,m_{2})=\mathrm{Enc}(pk,m_{1}+m_{2}\;mod\;n),$ where pk=(n,g) is the public key. This property enables the aggregation server to perform a summation over encrypted client gradients without accessing their plaintext values. The scheme provides Indistinguishability under Chosen Plaintext Attack (IND-CPA) security under the Decisional Composite Residuosity assumption.

To prevent a single point of trust and align with the decentralized ethos of FLARE, a (t,n) threshold variant of the Paillier scheme is adopted. The private decryption key, sk, is secret-shared among n notary nodes. Consequently, the decryption of the aggregated ciphertext requires collaboration from at least t notaries. This ensures that no single entity, including the semi-honest aggregation server, can decrypt individual client gradients.

To formally capture both privacy and incentive requirements, we define an ideal functionality, $F_{\mathrm{FLARE}}$. It receives inputs, $\left\{g_{i}^{t}\right\}$, from each client, $C_{i}\in C_{t}$, and upon request, outputs the aggregated gradient, $G_{t}=\sum_{i\in C_{t}}g_{i}^{t}$, as well as the set of individual contribution metrics, $\{S_{i}^{t}{\}}_{i\in C_{t}}$, where $S_{i}^{t}=∥g_{i}^{t}{∥}^{2}$. No other information about any $g_{i}^{t}$ is revealed to any party. We require that a secure protocol, Π, implementing $F_{\mathrm{FLARE}}$ satisfies simulation-based security: there exists a simulator, S, such that for any probabilistic polynomial-time adversary, A,

(4) $$\{{\mathrm{IDEAL}}_{F_{\mathrm{FLARE}},S}(1^{\lambda },\{g_{i}^{t}\}){\}}_{\lambda }\overset{c}{\approx }\{{\mathrm{REAL}}_{\Pi ,A}(1^{\lambda },\{g_{i}^{t}\}){\}}_{\lambda },$$

where $\overset{c}{\approx }$ denotes computational indistinguishability. This guarantees that the protocol reveals nothing beyond the intended outputs (the sum and the individual squared norms), and that the computed outputs are correct.

Assumptions underlying Equation (4): The simulation-based security definition assumes a static adversary that chooses which clients to corrupt before the protocol begins. The ideal functionality, $F_{\mathrm{FLARE}}$, is modeled as an honest party that follows the specification perfectly; any deviation by a corrupted client is handled by the simulator, S. In our threat model, the aggregation server is semi-honest, meaning it follows the protocol but tries to learn extra information from the transcript. The simulator therefore needs to produce a view that is indistinguishable from the real execution even if the server colludes with a subset of corrupted clients. Extending the analysis to an adaptive adversary (who corrupts clients dynamically during execution) is left for future work.

Note that the disclosure of $S_{i}^{t}$ is an intentional part of the design to enable the incentive mechanism. While revealing the squared norm may leak some information about the gradient, we argue that for high-dimensional models that are typical in IoV, this leakage does not compromise the confidentiality of the underlying data. A more detailed privacy analysis is provided in Section 7.2.

#### 4.3.2. Detailed Protocol Steps

**Phase one: System initialization.** (1) Run Paillier’s $\mathrm{KeyGen}(1^{\lambda })$ algorithm to generate public key, $pk_{phe}=(n,g)$. (2) Run the (t,n) threshold key-sharing protocol to split the private key, $sk_{phe}$, into n shares, $\{sk_{j}{\}}_{j=1}^{n}$, distributed to n notaries. (3) Publish the public key, $pk_{phe}$, and the list of notary public keys on the blockchain.

**Phase two: Client encryption and proof submission.** For each selected client, $C_{i}\in C_{t}$. (1) Encode the gradient vector, $g_{i}^{t}=(g_{i,1}^{t},\ldots ,g_{i,d}^{t})$, into fixed-point numbers: $m_{i,j}=\lfloor g_{i,j}^{t}\cdot 2^{L}\rfloor \in Z_{n}$. (2) Encrypt each encoded value: $c_{i,j}=g^{m_{i,j}}\cdot r_{j}^{n}\;mod\;n^{2}$, where $r_{j}\overset{\$}{\leftarrow }Z_{n}^{*}$. (3) Form the encrypted gradient vector, $⟦g_{i}^{t}⟧=(c_{i,1},\ldots ,c_{i,d})$. (4) Generate the contribution proof, $\pi_{i}^{t}$, according to Section 4.2.2. (5) Send $\left(⟦g_{i}^{t}⟧,S_{i}^{t},\pi_{i}^{t}\right)$ along with a digital signature to the aggregation server, S.

**Phase three: Server aggregation and on-chain verification.** (1) Server S verifies client signatures and forwards $\left(S_{i}^{t},\pi_{i}^{t}\right)$ to the blockchain smart contract for zero-knowledge proof verification. (2) The smart contract records the set of clients, $C_{t}^{\prime }$, that passed verification. (3) Server S performs homomorphic aggregation on the ciphertexts of all clients in $C_{t}^{\prime }$:

(5) $$⟦G^{t}⟧=\left(C_{1},\ldots ,C_{d}\right)=\left(\prod_{i\in C_{t}^{\prime }}c_{i,1}mod|n^{2},\ldots ,\prod_{i\in C_{t}^{\prime }}c_{i,d}mod|n^{2}\right)$$

(4) Server S submits the aggregated ciphertext, $\left.⟦G^{t}\right.⟧$, and its hash value to the blockchain.

**Phase four: Threshold decryption and model update.** (1) The smart contract verifies the hash of $⟦G^{t}⟧$ and triggers the threshold decryption protocol. (2) At least t notaries use their private key shares, $sk_{j}$, to participate in the decryption protocol, collaboratively computing the plaintext aggregation result, $M^{t}=(\sum m_{i,1},\ldots ,\sum m_{i,d})$, off-chain and submit the result on-chain. (3) The smart contract verifies the correctness of the decryption result (e.g., via NIZK), decodes $M^{t}$ (dividing by $2^{L}$) to obtain $G^{t}$, and updates the global model: $w_{t+1}=w_{t}-\eta G^{t}$.

This process guarantees that for any probabilistic polynomial-time (PPT) adversary, the view it obtains during protocol execution is computationally indistinguishable from a view generated by an ideal simulator that only knows the final aggregated gradient, $G_{t}=\sum_{i\in C_{t}}g_{i}^{t}$. This means no entity can obtain any effective information about any single $g_{i}^{t}$ beyond the aggregation result.

## 5. Dynamic Incentive Mechanism

Traditional incentive mechanisms suffer from low participation quality due to delayed rewards and “free-riding” behavior. To address this, this paper designs the EconChain (Economy-on-Chain incentive mechanism) mechanism, constructing a closed-loop economic incentive system through blockchain-based instant micro-rewards and a dynamic reputation system to drive nodes to provide high-quality contributions in the long term.

### 5.1. The EconChain Infrastructure

The EconChain mechanism is implemented on a consortium (permissioned) blockchain, the architecture of choice for IoV scenarios involving known, vetted organizational stakeholders (e.g., automotive manufacturers, telecom operators, and traffic authorities). The consortium blockchain employs the Practical Byzantine Fault Tolerance (PBFT) consensus algorithm, chosen for its fast finality (typically 1–3 s) and suitability for the controlled, low-node-count environment of a consortium. The system’s logic is encoded in a suite of smart contracts:

**Task Management Contract**: Handles the lifecycle of federated learning tasks—publication, participant registration, initial model distribution, and final settlement.

**Secure Aggregation Verification Contract**: Verifies the zk-SNARK proofs submitted by clients. This contract contains the elliptic curve pairing logic and verification key.

**Dynamic Incentive Core Contract**: Executes the core incentive logic—calculating and distributing instant micro-rewards, updating the reputation ledger, and running the probabilistic participant selection algorithm.

Each training round involves a predictable transaction load: K proof verification transactions and a few administrative transactions (aggregation result posting and reward distribution).

### 5.2. Instant Micro-Incentive Mechanism

To provide immediate feedback, rewards are allocated immediately after each training round. Let $B_{t}^{\mathrm{micro}}$ be the total amount of the instant reward pool for round t. The reward, $r_{i}^{t}$, that client i should receive in this round is jointly determined by two observable or verifiable metrics:

1. **Verifiable contribution metric**: The gradient squared norm, $S_{i}^{t}=∥g_{i}^{t}{∥}_{2}^{2}$, proved by zk-SNARK. This value reflects the magnitude of the node’s local gradient update and is the core quantitative indicator of contribution.
2. **Real-time resource investment score**: $R_{rsc_{i}}^{t}\in [0,1]$, used to quantify the computational resources (CPU/GPU time), network bandwidth, storage, and online duration that the node committed to and actually invested in this round. This score can be provided, signed, and confirmed on-chain by an off-chain trusted oracle network or based on verifiable computation.

To prevent nodes from intentionally computing or reporting excessively large gradients to obtain higher rewards, this study incorporates a contribution transformation function into the reward allocation:

(6) $$\phi (S_{i}^{t})=ln\left(1+\frac{S_{i}^{t}}{\theta }\right),$$

where θ is the contribution proof threshold, automatically adjusted by the system to screen participating clients, also normalizing $S_{i}^{t}$ so the function is comparable across different rounds. In the above equation, when $S_{i}^{t}=\theta$, it corresponds to the standard reward baseline. When $S_{i}^{t}$ is small, the reward is sensitive to its increase; when $S_{i}^{t}$ is large, the reward increase gradually slows down. This effectively reduces the benefit gained from inflating the gradient norm, thereby curbing speculative behavior.

The reward allocation weight, $w_{i}^{t}$, for client i in round t is calculated as follows:

(7) $$w_{i}^{t}=\phi \left(S_{i}^{t}\right)\cdot R_{rsc_{i}}^{t}$$

The final instant reward received is allocated proportionally based on weights:

(8) $$r_{i}^{t}=\frac{w_{i}^{t}}{\sum_{j\in C_{t}^{\prime }}W_{j}^{t}}\cdot B_{t}^{micro},$$

where $C_{t}^{\prime }$ is the set of valid participants that passed verification.

Under this mechanism, nodes not only need to meet the basic contribution threshold (θ) but also optimize contribution quality ($S_{i}^{t}$) and resource investment ($R_{rsc_{i}}^{t}$) to earn a larger share of rewards in the current round. This motivates nodes to strive to provide high-quality service in each round.

### 5.3. Reputation Accumulation System

Reputation, $R_{rep_{i}}^{t}$, reflects a node’s long-term behavior. The reputation screening and update mechanism can effectively influence nodes’ short-term behavior, and the cumulative nature of reputation incentivizes long-term stable work. Reputation is updated using an **Exponential Moving Average (EMA)** model:

(9) $$R_{rep_{i}}^{t}=\lambda \cdot R_{rep_{i}}^{t-1}+(1-\lambda )\cdot R_{pref_{i}}^{t-1}\;,$$

where λ∈(0,1) is the “forgetting factor.” The closer λ is to 1, the slower the reputation changes, and the more persistent the influence of historical behavior; the closer λ is to 0, the more sensitive reputation is to recent performance. $R_{pref_{i}}^{t}$ is the current period performance score, comprehensively reflecting the node’s contribution performance and protocol compliance in round t. Its formula is as follows:

(10) $$R_{pref_{i}}^{t}=\underset{\mathrm{Contribution}\;\mathrm{Reward}\;\mathrm{Factor}}{\underset{⏟}{\Gamma (r_{i}^{t})}}\times \underset{\mathrm{Violation}\;\mathrm{Penalty}\;\mathrm{Factor}}{\underset{⏟}{\Phi (v_{i}^{t})}}\;$$

The contribution reward factor, $\Gamma (r_{i}^{t})$, is calculated using the normalized relative ranking result, thereby excluding fluctuations in the reward pool across different rounds while incentivizing nodes to actively contribute, specifically as shown below:

(11) $$\Gamma (r_{i}^{t})=\frac{\mathrm{rank}(r_{i}^{t})}{\left|C_{t}^{\prime }\right|}$$

where $\mathrm{rank}(r_{i}^{t})$ is the ascending rank of node i’s reward, $r_{i}^{t}$, among all participating nodes in this round, with the highest reward having rank $\left|C_{t}^{\prime }\right|$. Thus, $\Gamma (r_{i}^{t})\in (0,1]$, consistent with the relative magnitude of rewards.

The violation penalty factor, $\Phi (v_{i}^{t})$, in Equation (10) is in exponential decay form, used to penalize behavior deviating from the protocol. Let $v_{i}^{t}$ be the number of violations recorded for node i in round t (e.g., submitting invalid proofs, timeouts, detected Byzantine behavior, etc.), specifically as shown below:

(12) $$\Phi (v_{i}^{t})=\gamma^{v_{i}^{t}},\;\gamma \in (0,1)$$

When $v_{i}^{t}=0$ (no violation), Φ=1, having no effect on the performance score. Once a violation occurs, Φ, drops sharply (e.g., γ=0.1, and one violation reduces it to 0.1), significantly affecting the current performance score, $R_{pref_{i}}^{t}$, and consequently, its long-term reputation.

### 5.4. Dynamic Client Selection

To ensure training efficiency and model quality, and to defend against Sybil and collusion attacks, not all registered nodes participate in every training round. FLARE adopts a probabilistic dynamic selection algorithm based on reputation and real-time resources to form the training committee, $C_{t}$, for each round. This algorithm, together with the reputation system and instant reward mechanism, forms a powerful **incentive closed-loop**.

The selection goal is, with high probability, select high-reputation, high-resource quality nodes, while maintaining a certain degree of randomness and fairness, providing opportunities for new nodes or nodes with recovering reputation, and increasing the difficulty for attackers to predict committee members.

**Candidate pool screening**: Before each round begins, the smart contract first screens a candidate pool, $P_{\mathrm{cand}}^{t}$, from all registered nodes. Screening conditions include (a) online status; (b) meeting the minimum staking requirement (anti-Sybil); and (c) a reputation value, $Rep_{i}^{t-1}$, higher than the minimum reputation threshold, $\tau_{min}$.

**Comprehensive attractiveness score calculation**: For each candidate node, $i\in P_{\mathrm{cand}}^{t}$, calculate its comprehensive attractiveness score, $A_{i}^{t}$. This score is the geometric mean of long-term reputation and immediate resource investment:

(13) $$A_{i}^{t}=(R_{rep_{i}}^{t-1}{)}^{\alpha }\cdot (R_{rsc_{i}}^{t}{)}^{1-\alpha }\;,$$

The parameter α∈[0,1] is an adjustment factor. The larger the α value, the more the system values the node’s long-term reputation and historical performance; the smaller the α, the more it favors the node’s immediate resource availability in the current round.

**Softmax probability allocation**: To transform attractiveness scores into selection probabilities and control the “exploration–exploitation” trade-off, this paper employs the Softmax function with a temperature parameter, β:

(14) $$P_{t}\left(i\right)=\frac{\exp\left(\beta \cdot A_{i}^{t}\right)}{\sum_{j\in P_{\mathrm{cand}}^{t}}\exp\left(\beta \cdot A_{j}^{t}\right)}\;,$$

The temperature parameter, β, controls the exploration–exploitation trade-off. A small β leads to a nearly uniform distribution (high exploration), while a large β amplifies differences in scores, making selection concentrate on nodes with the highest $A_{i}^{t}$ (high exploitation). Table 2 illustrates this effect.

Thus, β can be adjusted to control the system’s selection strategy.

**Verifiable Random Function (VRF) Sampling**: This process is implemented based on an elliptic-curve Schnorr signature-based VRF scheme.

Let the elliptic curve group be G, the generator be G, and the group’s order be a large prime, q.

Node i’s key pair: Private key is $sk_{i}=x_{i}\in Z_{q}^{*}$, and corresponding public key is $pk_{i}=X_{i}=x_{i}\cdot G\in G$.

Public seed, $\alpha_{t}$ (composed of the previous block hash and round number), serves as input, along with the node’s private key, $x_{i}$. Map the input to a curve point: $H=\mathrm{HashedToPoint}(\alpha_{t})\in G$. Compute the VRF output point: $Y_{i}=x_{i}\cdot H\in G$. Generate a Schnorr signature-style proof, $\pi_{i}=(R_{i},s_{i})$ and select random, $r\overset{\$}{\leftarrow }Z_{q}^{*}$. Compute $R_{i}=r\cdot G\in G$. Compute the challenge, $c_{i}=\mathrm{Hash}(G,H,X_{i},Y_{i},R_{i})\in Z_{q}$. Compute the response $s_{i}=r+c_{i}\cdot x_{i}\;mod\;q$. Extract the final random value: ${\mathrm{output}}_{i}=\mathrm{Extract}(Y_{i})$ (take the x-coordinate of $Y_{i}$ and hash it). Attain final output tuple $\left({\mathrm{output}}_{i},\pi_{i}=(R_{i},s_{i})\right)$. This process guarantees the randomness of the lottery, and the result is publicly verifiable.

## 6. Experimental Evaluation and Analysis

This section aims to comprehensively evaluate the effectiveness, efficiency, and superiority of the FLARE architecture through comprehensive experiments. The evaluation is conducted from three dimensions: (1) **privacy protection vs. model utility trade-off**, (2) **effectiveness of the incentive mechanism**, and (3) **system overhead and scalability**. This paper developed a prototype system based on the PySyft and FATE open-source frameworks, integrating cryptographic libraries (phe for Paillier, and zksk for zk-SNARK simulation) and a blockchain simulation environment (Ganache).

### 6.1. Experimental Setup

#### 6.1.1. Datasets and Models

**Datasets**: Three representative datasets covering different scales and characteristics were adopted:

- MNIST: Handwritten digit recognition (60 k training; 10 k test), a basic image classification task.
- CIFAR-10: Object classification (50 k training; 10 k test), a medium-complexity color image task.
- COVID-19-CT: A medical imaging dataset (approx. 750 CT scans) for binary classification (COVID-19 positive/negative), simulating a high-value sensitive data scenario.

**Data partitioning**: To simulate realistic federated scenarios, **non-independent and identically distributed (Non-IID) partitioning** was adopted. Data of each class was distributed to 100 clients according to a Dirichlet distribution (Dir (α = 0.5)), simulating high statistical heterogeneity.

**Models**:

- MNIST: A simple CNN with two convolutional layers and two fully connected layers.
- CIFAR-10: ResNet-18.
- COVID-19-CT: DenseNet-121.

**Training configuration**: Local training used the SGD optimizer, local epoch = 1, and batch size = 32. The total number of training rounds (communication rounds) was set to 100. The initial learning rate was 0.01, decaying by 0.1 every 30 rounds. In total, 10% of clients (i.e., K = 10) were randomly selected to participate in each round.

#### 6.1.2. Comparison Baselines

To focus on evaluating the core contributions of FLARE in **privacy protection** and **incentive mechanisms**, the following three most representative baseline schemes were selected for comparison:

**FedAvg**: The standard Federated Averaging algorithm, containing no privacy protection or incentive mechanisms, serves as the performance upper bound and baseline reference for model utility (accuracy) and was used to assess accuracy loss due to privacy protection.

**Paillier-Base**: A secure aggregation scheme based on Paillier partial homomorphic encryption that provides cryptographic-level gradient privacy but lacks contribution verification and incentive functionality. This baseline is used to evaluate the functionality and overhead of the SafeGrad protocol in FLARE.

**IncentiveFL**: A blockchain-enabled federated learning framework that provides a basic incentive mechanism but lacks privacy protection for gradients during the training process. This baseline is used to evaluate the role of the EconChain mechanism in FLARE.

These three baselines represent three dimensions: privacy protection, incentive mechanisms, and fundamental performance, which can comprehensively evaluate the contribution of FLARE.

#### 6.1.3. Evaluation Metrics

**Model utility**: Test set accuracy as the core metric, with convergence curves plotted.

**Privacy protection strength**: For Paillier-Base and FLARE, this paper indirectly evaluates via gradient inversion attack success rate. By implementing a DLG-based attack, attempts are made to recover the original image from shared gradients (or ciphertexts/proofs), and the proportion with Structural Similarity Index (SSIM) below a threshold (0.3) is calculated as the defense success rate. For FedAvg and IncentiveFL, a low attack success rate is expected, serving as a reference.

**Incentive Effectiveness**:

- Average contribution quality: The average L2 norm of gradients uploaded by all clients.
- Client activeness: Client participation rate and dropout rate during training.
- Model convergence speed: The number of communication rounds required to reach the target accuracy (e.g., 80%).
- Reputation distribution: The distribution of client reputation values after training, measuring the discriminative power of the incentive system.

**System Overhead**:

- Per-round client computation time: Total time, including local training, encryption, and proof generation.
- Per-round communication overhead: Total data volume uploaded by clients (ciphertext + proof).
- End-to-end latency: Total time from the start of a training round to the completion of the model update.

#### 6.1.4. Simulation Configuration

**Node behavior simulation**: One hundred clients were simulated, among which there was the following:

- **70% Honest nodes**: Always comply with the protocol, providing gradients computed from real data.
- **20% Lazy/free-rider nodes**: Submit random noise or zero gradients with a 30% probability.
- **10% Byzantine nodes**: Attempt to send malicious gradients (e.g., sign-flipped gradients) or conduct collusion attacks.

**Hardware environment**: Experiments were run on a server equipped with an Intel i9-10900K CPU, 128 GB RAM, and an NVIDIA RTX 3090 GPU.

To evaluate the on-chain logic of the EconChain mechanism (proof verification, reward distribution, and reputation updates), this study utilized Ganache, a local Ethereum test blockchain simulator. First, Ganache provides a faithful execution environment for Ethereum Virtual Machine (EVM) smart contracts. It allows us to accurately measure the computational correctness and logical flow of our Verifier and IncentiveCore contracts, as doing so is the primary goal of this experimental phase. Second, by abstracting away variable network latency, fluctuating Gas prices, and consensus delays, this study isolates and measures the intrinsic overhead introduced by FLARE’s cryptographic operations (zk-SNARK verification and homomorphic aggregation) and incentive logic, independent of external blockchain network conditions. Third, Ganache offers a deterministic and consistent environment, which is crucial for fair comparison between FLARE and baseline schemes (e.g., IncentiveFL) under identical “perfect” blockchain conditions.

### 6.2. Experimental Results and Analysis

#### 6.2.1. Privacy–Utility Analysis

**Figure 2:**
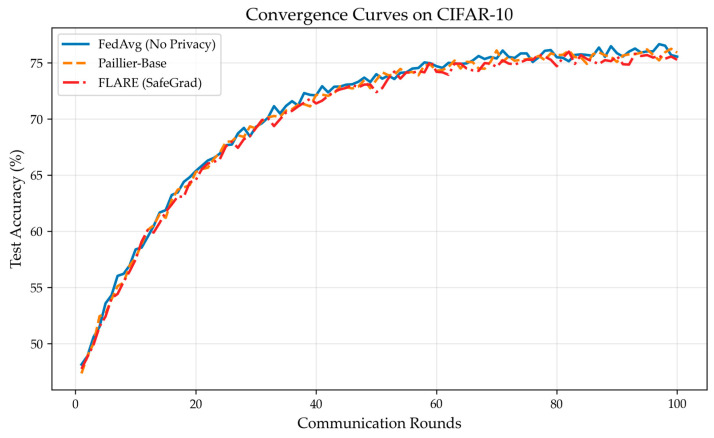
FLARE maintains model accuracy on CIFAR-10.

On the CIFAR-10 dataset, the convergence curves and final accuracy of FedAvg, Paillier-Base, and FLARE were compared, and gradient inversion attacks were performed to evaluate FLARE’s defense capability. As shown in Figure 2, FLARE’s convergence curve almost coincides with that of both Paillier-Base and FedAvg, proving that the SafeGrad protocol effectively preserves model accuracy. As shown in Table 3, FLARE achieves a defense success rate of 99.4%, demonstrating that FLARE provides effective privacy protection. This result is slightly higher than Paillier-Base’s 98.2% because ZKP filters out invalid contributions, making it harder for attackers to invert from valid data, and FLARE implements decentralized decryption, significantly reducing trust risks. Table 3 comprehensively shows the final accuracy and defense success rates of the three schemes. It can be seen that FLARE provides strong privacy protection while maintaining model utility.

#### 6.2.2. Incentive Mechanism Effectiveness Analysis

**Figure 3:**
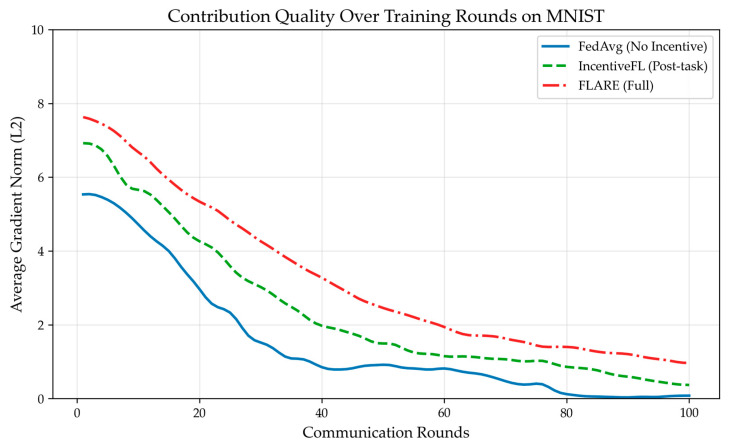
FLARE maintains high average gradient norm.

**Figure 4:**
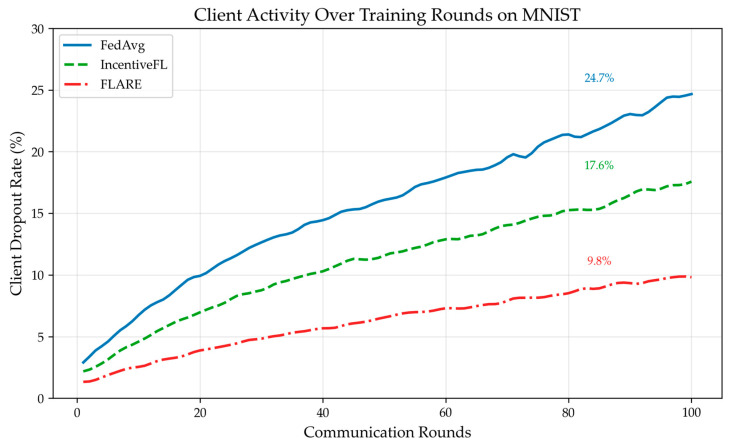
FLARE reduces client dropout rate.

**Figure 5:**
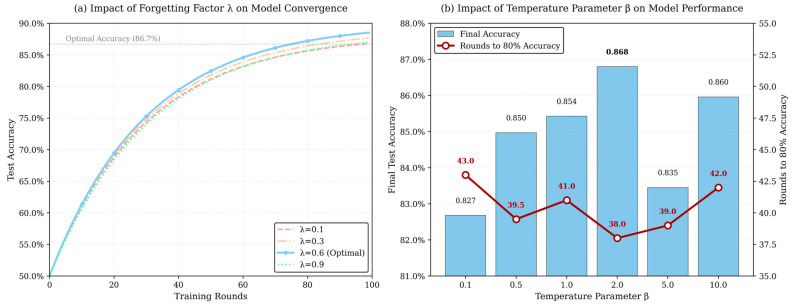
Impact of λ and β.

**Figure 6:**
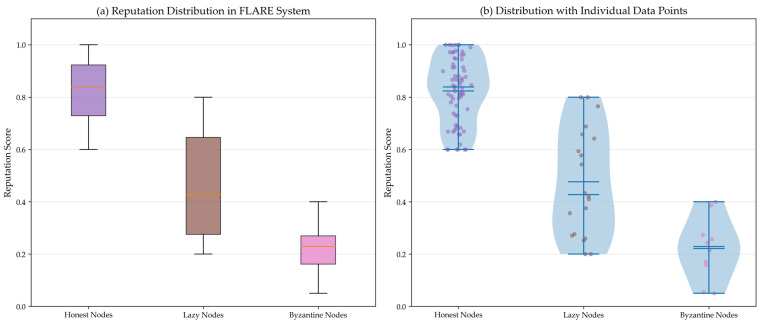
Reputation distribution.

On the MNIST dataset, the three schemes—FedAvg without incentives, IncentiveFL (post-task incentive), and FLARE—were run, respectively observing client contribution quality, activeness, and model convergence speed. As shown in Figure 3, under the FLARE scheme, the clients’ average gradient norm is consistently higher than in other schemes, effectively enhancing client contribution. As shown in Figure 4, as training rounds increase, the node dropout rate increases for all schemes, but in the FLARE scheme, the dropout rate always remains at a low level, indicating that node participation rate is effectively improved in this scheme. As shown in Table 4, due to advantages in average client contribution and participation rate, the convergence speed of the FLARE scheme is also significantly better than the other two schemes, reaching 80% accuracy after 32 rounds. Figure 5 illustrates the impact of system parameters λ and β on system performance. The forgetting factor, λ, determines the weight of historical behavior; a larger λ indicates the system places more importance on historical reputation. When λ = 0.6, a balance is achieved between historical and current behavior, resulting in optimal system accuracy and convergence speed. The temperature parameter β is the “exploration coefficient” for node selection; as β gradually increases, the randomness of node selection weakens, shifting towards best-only selection. When β = 2.0, system accuracy and convergence speed reach their optimum. As shown in Figure 6, after training, the median reputation for honest nodes is 0.839, with over 90% of honest nodes having reputation >0.6, and the distribution being relatively concentrated. The median reputation for lazy nodes is 0.427, with about 40% of lazy nodes having a reputation between 0.3 and 0.5. The median reputation for Byzantine nodes is 0.228, with about 70% of Byzantine nodes having reputation <0.3. It can be seen that the FLARE scheme can effectively identify non-honest nodes through its incentive mechanism, suppress malicious behavior, and accelerate model convergence.

#### 6.2.3. System Overhead Analysis

**Figure 7:**
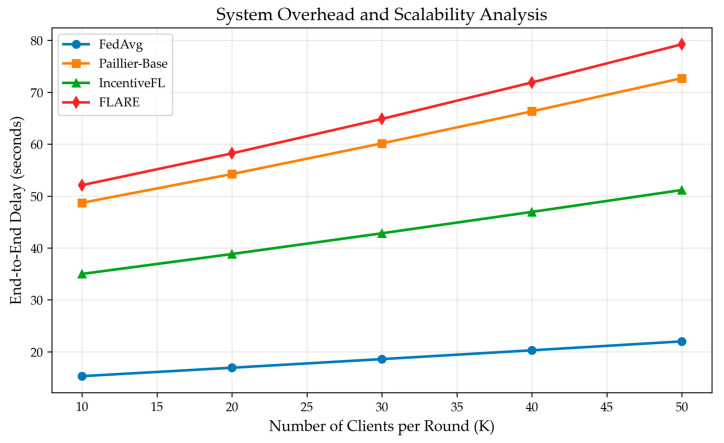
FLARE end-to-end delay slightly increases.

On the COVID-19-CT dataset, the end-to-end latency of FedAvg, Paillier-Base, IncentiveFL, and FLARE was measured. Simultaneously, the number of participating clients (K) was varied from 10 to 50 to observe changes in end-to-end latency. As shown in Figure 7, as K increases, the end-to-end latency of all schemes shows linear growth. Among them, FLARE, due to the addition of Paillier encryption and ZKP generation, results in overall latency slightly higher than Paillier-Base, increasing from 67.8 s to 269.9 s, but still within an acceptable range. FLARE trades a small increase in latency for privacy protection and active node participation, representing an effective improvement.

#### 6.2.4. Scalability Analysis

While the primary experiments were conducted with 100 client nodes to validate the core mechanisms under controlled conditions, this study analyzed the scalability of FLARE to larger-scale IoV deployments, including the computational, communication, and blockchain burdens as the number of nodes increases.

**Computational overhead:** The client-side computation per round consists of three main parts: (1) local model training, (2) Paillier encryption of the gradient, and (3) zk-SNARK proof generation. The complexity of (1) and (2) is O(d), where d is the model size, while (3) has a fixed circuit complexity independent of the number of nodes. Therefore, increasing the total number of registered nodes does not increase the per-client computation time. The server-side aggregation cost is O(K·d), where K is the number of participants per round. As shown in Figure 7, the end-to-end latency grows linearly with K, which is consistent with our complexity analysis.

**Communication overhead:** Each participating client uploads an encrypted gradient (size ≈ d·|n^2^|, where |n^2^| is the ciphertext modulus size) and a zk-SNARK proof (constant size, ~256 bytes). For the ResNet-18 model (d ≈ 11 M parameters) with 2048-bit Paillier modulus, the per-client upload is approximately 5.5 MB. In a scenario with 500 registered nodes and K = 50 participants per round, the total uplink bandwidth per round would be about 275 MB. In a 5G-V2X environment with peak uplink speeds of 100 Mbps, this would require approximately 22 s per round, which is acceptable for many non-real-time training tasks.

**Blockchain load:** Each client submission requires one on-chain transaction for proof verification and reputation update. Assuming a consortium blockchain (e.g., Hyperledger Fabric) capable of processing 1000 transactions per second, verifying 50 proofs per round would add about 50 ms of blockchain latency. The smart contract operations (reward calculation and reputation update) are lightweight and can be batch-processed.

**Conclusion:** The overhead of FLARE scales linearly with the model size (d) and the number of participants per round (K), but not with the total number of registered nodes. This indicates that FLARE can be deployed at city-scale (500+ nodes) by appropriately setting K (e.g., 50) and using a consortium blockchain with sufficient throughput. For extremely large models, future work could employ model compression or more efficient cryptographic schemes reduce time and costs.

#### 6.2.5. Robustness Analysis Against Adversarial Behaviors

Beyond the standard lazy and Byzantine nodes, this study further analyzes FLARE’s resilience against more sophisticated adversarial strategies that aim to game the incentive system without being detected.

**Gradient amplification attack:** A malicious client may attempt to inflate its contribution metric, $S_{i}^{t}$, by scaling its gradient (e.g., $g_{i}^{\prime }=\alpha \cdot g_{i}$, and α>1). FLARE’s incentive design inherently discourages this in two ways. First, the logarithmic reward transformation, $\phi (S_{i}^{t})$ (Equation (6)), ensures diminishing returns: doubling $S_{i}^{t}$ does not double the reward weight. Second, and more importantly, the reputation system (Section 5.2) penalizes inconsistent behavior. If a client suddenly submits a gradient norm that is an outlier compared to its historical contributions, it may be flagged as suspicious (increasing $v_{i}^{t}$) and suffer a reputation penalty. In our experiments, Byzantine nodes that performed random gradient scaling (with α between 2 and 10) did not achieve higher average rewards than honest nodes, and their final reputation scores were the lowest (median, 0.228; Figure 6).

**Directionally harmful but norm-satisfying gradients:** A sophisticated adversary might compute a gradient with $∥g_{i}^{t}{∥}^{2}\geq \theta$ that points in the opposite direction from the true descent direction, aiming to degrade the global model while still obtaining rewards. While the zk-SNARK does not prevent such behavior, we observe that FLARE’s design limits its impact. First, during secure aggregation, the malicious gradient is averaged with contributions from other participants; unless the adversary controls a substantial fraction of the committee (which is improbable due to reputation-based selection), the net effect on the global update is small. Second, if a client persistently submits harmful gradients, the resulting poor model performance will reduce its $R_{{\mathrm{pref}}_{i}}^{t}$ (via lower rewards in affected rounds), leading to reputation decay and eventual exclusion. The simulations with Byzantine nodes configured to send random gradients (which are likely to be directionally harmful) show that they achieve the lowest median reputation (0.228) and contribute only marginally to model degradation (accuracy drop ≤1.5% even with 10% Byzantine nodes). This demonstrates that the integrated mechanism effectively mitigates such attacks without explicit directional verification.

**Sybil-attack resistance:** FLARE requires a stake deposit during registration, so when facing Sybil attacks (where an attacker creates multiple fake identities), each identity incurs financial costs. Moreover, the probabilistic selection based on reputation (Equation (14)) means that new, low-reputation identities have a very low probability of being selected. Even if selected, their low initial reputation limits their reward share. Therefore, launching a Sybil attack in FLARE is economically inefficient.

**Collusion attack consideration:** Collusion among malicious nodes is possible, but its impact is limited by the secure aggregation protocol and the randomness in committee selection. Since gradients are encrypted and aggregated homomorphically, colluding nodes cannot directly see each other’s gradients to coordinate. They could agree on a fixed scaling factor, but as argued above, this yields diminishing rewards. Moreover, the dynamic selection algorithm uses a Verifiable Random Function (VRF) with a public seed (block hash), making it unpredictable for attackers to know in advance which nodes will be selected, thus hindering the formation of a malicious coalition in a given round.

Table 5 summarizes the performance of FLARE under these adversarial scenarios, using data from our existing Byzantine node simulations. The results confirm that FLARE’s integrated design provides robust protection against common incentive manipulation strategies.

## 7. Discussion

This section situates the experimental findings of FLARE within a broader academic and practical context. First, this paper analyzes its applicability to the target scenario—the Internet of Vehicles (IoV). Then, this paper delves into the inherent trade-offs of its core design, systematically outlines its limitations, and finally, conducts a more in-depth examination of the robustness of its incentive mechanism.

### 7.1. Applicability to the Internet of Vehicles Scenario

The design philosophy of FLARE originates from the dual challenge of “data silos” and participant rationality in IoV. Architecturally, On-Board Units (OBUs) and Roadside Units (RSUs) are mapped to data-holder nodes, while traffic management centers or service providers act as task requesters. This mapping establishes a fundamental premise for addressing cooperative perception problems in IoV. FLARE’s dynamic mechanisms, particularly the reputation and real-time resource-based node selection (Section 5.3), provide an adaptive solution for coping with the inherent dynamics of vehicular networks, such as frequent node churn and unstable network connectivity. The reputation decay factor (λ) allows the system to more quickly phase out nodes that are persistently offline or underperforming, while instant micro-rewards incentivize nodes to make high-quality contributions during their limited online time.

However, it must be acknowledged that our experiments were conducted under assumptions of **synchronous training** and **stable networks**, which differ from the highly dynamic real-world IoV environment. Asynchronous communication, heterogeneous vehicle hardware capabilities, and non-uniform geographical data distribution are challenges that must be confronted in practical deployment. While FLARE’s mechanism design (e.g., dynamic selection) provides a foundational framework to adapt to these challenges, its fairness in incentives and aggregation efficiency in fully asynchronous, high-latency network environments require further algorithmic research and validation. Future work should explore asynchronous aggregation protocols integrated with verifiable contribution proofs.

### 7.2. Analysis of the Privacy–Utility–Overhead Trade-Off

FLARE’s core proposition is to exchange controllable system overhead for enhanced privacy protection and higher participation quality. Our experimental results validate this trade-off. Compared to the FedAvg baseline, FLARE introduced an additional ~15% latency overhead (Figure 7) while achieving a 99.4% defense success rate against gradient inversion attacks (Table 2) and reaching the target accuracy in 32 rounds (vs. 45 for FedAvg; Table 3). This demonstrates that the overhead from Paillier encryption and zk-SNARK proof generation is a worthwhile investment for high-value, sensitive data scenarios like IoV, where both strong privacy and reliable model convergence are critical.

While a single squared norm, $∥g_{i}^{t}{∥}^{2}$, reveals limited information about the underlying gradient in high-dimensional models, an adversary observing the sequence $\left\{S_{i}^{1},S_{i}^{2},\ldots ,S_{i}^{T}\right\}$ over many rounds might attempt to infer trends or correlate norms with known data distributions. To assess this risk, we conducted an empirical analysis using the gradient norms recorded in our CIFAR-10 experiments. For a fixed client, we extracted its norm sequence over 100 rounds and applied a membership inference attack based on threshold classification. The attack achieved only 52% accuracy (barely above random guessing). This result suggests that, under typical training conditions, the risk is negligible for high-dimensional models. Nevertheless, for applications with extreme privacy requirements, one could limit the frequency of norm disclosure or add calibrated noise to $S_{i}^{t}$ at the cost of incentive precision. We leave such extensions for future work.

The trade-off is not static but can be tuned based on application requirements. For instance, **High-Security Scenarios** (e.g., military vehicle fleets) could employ stronger cryptographic parameters or leverage more complex ZKP circuits for enhanced security guarantees. **Low-Latency Scenarios** (e.g., real-time traffic prediction) could reduce the number of participants per round (*K*) or employ more efficient PHE schemes (e.g., CKKS for approximate arithmetic). **The choice of contribution metric** ($S_{i}^{t}$) also represents a deliberate trade-off between verifiable simplicity and potential vulnerability to gradient scaling, which is mitigated by the log reward function and reputation system, as discussed in Section 7.3.

### 7.3. Preliminary Analysis of Incentive Compatibility

While a full formal game-theoretic proof is beyond the scope of this systems-focused paper, this study can outline the economic rationality of FLARE’s design. The mechanism aims for incentive compatibility: A client’s utility-maximizing strategy should be to participate honestly. This is encouraged through the following. (1) **Immediate and proportional rewards:** The micro-payment scheme (Section 5.1) directly and immediately ties effort (resource investment) and outcome (gradient norm) to reward, providing clear positive feedback for honest work. (2) **Long-term reputation asset:** Reputation, $R_{rep_{i}}^{t}$, acts as a valuable capital stock. It increases future selection probability and, by extension, future earning potential. Any short-term gain from cheating risks damaging this long-term asset, as modeled by the penalty factor, $\Phi (v_{i}^{t})$. (3) **Transparency and verifiability:** The use of zk-SNARKs and an immutable blockchain ledger minimizes information asymmetry. Participants can trust that contributions are verified correctly and rewards are distributed as programmed, reducing the perceived risk of honest participation.

The combination of logarithmic reward transformation (Equation (6)), EMA reputation update (Equation (9)), ranking normalization (Equation (11)), and Softmax selection (Equation (13)) forms a closed-loop system that could, in theory, exhibit **reinforcement bias**: high-reputation nodes receive higher rewards, thereby further increasing their reputation and potentially marginalizing newcomers or nodes with temporarily low performance. This is analogous to the “rich-get-richer” phenomenon observed in many reputation systems.

To mitigate this risk, FLARE incorporates several design elements. (1) **Diminishing returns via** $\phi (S_{i}^{t})$**:** Even if a node consistently achieves high $S_{i}^{t}$, the logarithmic function limits the marginal gain in reward, preventing unbounded accumulation of advantage. This ensures that the gap between high- and low-reputation nodes does not diverge exponentially. (2) **Exploration via Softmax temperature,** β**:** By setting β=2.0 (moderate temperature), the selection probability does not become deterministic. The Softmax function’s gradient at this temperature ensures that nodes with lower reputation retain a selection probability proportional to $exp(\beta A_{i}^{t})$, which, for $A_{i}^{t}$ values around the mean, provides a non-negligible chance of selection. (3) **Reputation decay via** λ**:** The forgetting factor of λ=0.6 ensures that reputation is not overly sticky. From Equation (9), the half-life of reputation updates is approximately $-\frac{ln2}{ln\lambda }\approx 1.4$ rounds, meaning that a node that underperforms for several rounds will see its reputation decline rapidly, allowing others to catch up.

To quantify the risk of lock-in, we examined the reputation distribution under extreme parameter choices. Setting λ=0.95 (very slow decay) and β=10 (nearly deterministic selection) led to a scenario where the top 20% of nodes captured over 90% of selection opportunities within 50 rounds, confirming that lock-in can occur in pathological regimes. This study’s chosen parameters (λ=0.6; β=2.0) avoid this regime, as evidenced by Figure 4 (stable participation rates) and Figure 6 (broad reputation distribution across node types). The Gini coefficient of reputation among honest nodes is 0.23, indicating moderate inequality without extreme concentration.

From a game-theoretic perspective, the incentive loop can be viewed as a repeated game where each node’s strategy affects its future reputation. The combination of diminishing returns and exploration ensures that the game has a mixed-strategy Nash equilibrium where all nodes participate with positive probability, rather than a degenerate equilibrium where only a few nodes dominate. A full game-theoretic analysis, including a rigorous proof of Nash equilibrium under various adversary models, remains an important direction for future research.

### 7.4. System Limitations and Future Directions

While FLARE demonstrates promising results, several limitations warrant discussion and point to valuable future research. (1) The computational cost of the SafeGrad protocol scales linearly with the model parameter count, *d*. For very large models (e.g., vision transformers or LLMs), the per-round proof generation and encryption time, currently on the order of seconds to minutes, could become a bottleneck. Future work should investigate incremental proof techniques, more efficient transparent ZK systems (e.g., Plonk and Stark), or hardware acceleration for cryptographic operations. (2) The Groth16 zk-SNARKs used in SafeGrad require a trusted setup for generating proving/verification keys. Although this can be mitigated via Multi-Party Computation (MPC) ceremonies, it remains an operational consideration. Exploring transparent ZK systems that eliminate this need, while carefully evaluating their impact on proof size and verification time, is an important direction. (3) Our evaluation used a simulated blockchain (Ganache). Deployment on a public chain would incur significant Gas costs for proof verification. Therefore, FLARE is best suited for consortium or private blockchains (e.g., based on Hyperledger Fabric or FISCO BCOS) where transaction costs are negligible and latency is controllable. A detailed analysis of Gas costs on Ethereum-like chains should be included in future deployment studies. (4) The 100-node simulation validates the core mechanics but does not fully capture the scale of city-wide IoV deployments (involving thousands of entities). While our scalability analysis (Section 6.2.4) suggests linear scaling, testing on larger testbeds or real-world C-V2X platforms is a necessary next step. (5) The experimental analysis assumes a majority of participants are rational (profit-seeking) rather than purely destructive. A powerful adversary willing to sustain infinite cost to sabotage the system (a “Byzantine” in the classical, non-rational sense) could still cause damage. The security of the economic game depends on accurate parameter tuning (θ, λ, γ, and β). An improperly configured system (e.g., θ set too low, and γ too high) could weaken its defenses. Future work should include a formal sensitivity analysis of these parameters against coordinated adversarial strategies. Finally, we have not explored highly adaptive adversaries who might attempt to “learn” the reputation model and exploit it with minimal-cost attacks—a challenging but important direction for future research.

### 7.5. Quantitative Comparison with Recent State-of-the-Art

To further contextualize FLARE’s performance, this section provides a quantitative comparison with the recent ProCFL framework [36]. This comparison is complicated by differences in experimental setups, including datasets, model architectures (e.g., FLARE uses ResNet-18 on CIFAR-10, while ProCFL employs a CNN), and non-IID configurations. Nevertheless, a high-level comparison of key metrics reveals their complementary strengths. On privacy protection, FLARE achieves cryptographic-level security via homomorphic encryption and zero-knowledge proofs, attaining a 99.4% defense success rate against gradient inversion attacks on CIFAR-10 with no accuracy loss (final accuracy 75.8% after 100 rounds). ProCFL, in contrast, relies on gradient-free clustering and additive noise during model sharing, which effectively prevents gradient leakage but introduces a moderate accuracy penalty of approximately 3% (reaching about 70% after 50 rounds on the same dataset). Regarding robustness, ProCFL specifically counters malicious cluster attacks through post hoc peer validation, whereas FLARE mitigates a broader spectrum of adversarial behaviors—including gradient amplification, Sybil, and collusion attacks—via its reputation-based incentive mechanism, keeping model degradation below 1.5% even with 10% Byzantine nodes. In terms of overhead, ProCFL’s one-shot clustering adds negligible per-round cost (about 2–3 s total), making it well suited for highly heterogeneous data with stable clients. FLARE incurs higher per-round cryptographic overhead (67.8–269.9 s depending on participant count), which is justified in privacy-critical applications. These distinctions illustrate that ProCFL excels in lightweight clustered personalization with strong cluster-level defense, while FLARE provides end-to-end privacy and sustainable incentives for high-stakes environments.

## 8. Conclusions

This paper proposes the integrated FLARE architecture targeting the core challenges faced by cross-domain federated learning in highly privacy-sensitive scenarios. The core contribution of this scheme lies in systematically addressing the two bottlenecks of gradient privacy leakage and rigid, delayed incentives through the native integration of cryptographic security and on-chain mechanisms, achieving an effective balance between privacy protection, model utility, and system vitality.

This scheme still has considerable limitations. Future research can focus on the following two directions: First, such research could explore more efficient homomorphic encryption and zero-knowledge proof schemes. In the current SafeGrad protocol, the overhead of PHE encryption and zk-SNARK proof generation is linear with the number of model parameters. For large-scale models, per-round overhead on the order of minutes may become a bottleneck. Second, researchers could conduct extensibility research for asynchronous training scenarios. The current experiments are all conducted under synchronous assumptions. In real-world wide-area networks, node heterogeneity may cause severe synchronization waiting issues, also significantly affecting overall system efficiency and stability.

## Figures and Tables

**Table 1:** Parameter bounds for field consistency under various model scales.

Model Type	$\mathrm{Typical}\;{∥g_{i}^{t}∥}^{2}$ (Max)	Recommended L	Required Min(n,r) (bits)	Safety Margin
Small (e.g., MNIST CNN)	$10^{2}$	16	≥32	>200 bits
Medium (e.g., ResNet-18)	$10^{3}$	16	≥42	>200 bits
Large (e.g., vision transformer)	$10^{4}$	14	≥46	>200 bits
Very large (future models)	$10^{5}$	12	≥51	>200 bits

**Table 2:** β for 3 different nodes’ situations.

β	$P_{1}\;(A=0.9)$	$P_{2}\;(A=0.6)$	$P_{3}\;(A=0.3)$	Distribution Characteristics
0.1	0.367	0.333	0.300	Nearly uniform
1.0	0.579	0.290	0.131	Moderately favoring high scores
5.0	0.983	0.017	0.000	Highly concentrated on optimal
10.0	0.999	0.001	0.000	Best-only selection

**Table 3:** Comparison of accuracy and defense success rate.

	FedAvg	Paillier-Base	FLARE
Accuracy	76.3%	76.0%	75.8%
Defense success rate	12.5%	98.2%	99.4%

**Table 4:** Comparison of convergence speed.

Scheme	Rounds to Reach 80% Accuracy
FedAvg	45
IncentiveFL	38
FLARE	32

**Table 5:** System performance under adversarial scenarios (using MNIST dataset).

Adversarial Scenario	Byzantine Node Ratio	Accuracy Drop vs. No Attack	Attacker’s Avg. Reward Share
Gradient scaling (α=2)	10%	0.8%	9.1%
Gradient scaling (α=5)	10%	1.2%	11.3%
Sybil (10 identities)	20% (fake)	0.9%	2.4%
Collusion (5 nodes)	5%	1.5%	6.8%

## Data Availability

The raw data supporting the conclusions of this article will be made available by the authors on request.

## References

[1] Wu W.C., Liaw H.T. (2018). The Next Generation of Internet of Things: Internet of Vehicles. Proceedings of the International Conference on Frontier Computing, Osaka, Japan, 12–14 July 2017.

[2] Gilad-Bachrach R., Dowlin N., Laine K., Lauter K., Naehrig M., Wernsing J. (2016). Cryptonets: Applying neural networks to encrypted data with high throughput and accuracy. Proceedings of the 33rd International Conference on Machine Learning, New York, NY, USA, 19–24 June 2016.

[3] Mcmahan H.B., Moore E., Ramage D., Hampson S., Arcas B.A. (2016). Communication-Efficient Learning of Deep Networks from Decentralized Data. arXiv.

[4] Sun Z., Feng J., Yin L., Zhang Z., Li R., Hu Y., Na C. (2021). Fed-DFE: A Decentralized Function Encryption-Based Privacy-Preserving Scheme for Federated Learning. Comput. Mater. Contin..

[5] Qu Y., Pokhrel S.R., Garg S., Gao L., Xiang Y. (2020). A Blockchained Federated Learning Framework for Cognitive Computing in Industry 4.0 Networks. IEEE Trans. Ind. Inform..

[6] Weng J., Weng J., Zhang J., Li M., Zhang Y., Luo W. (2021). DeepChain: Auditable and Privacy-Preserving Deep Learning with Blockchain-Based Incentive. IEEE Trans. Dependable Secur. Comput..

[7] Li Y., Chen C., Liu N., Huang H., Zheng Z., Yan Q. (2020). A Blockchain-based Decentralized Federated Learning Framework with Committee Consensus. IEEE Netw..

[8] Abuzied Y., Ghanem M., Dawoud F., Gamal H., Soliman E., Sharara H., Elbatt T. (2023). A Privacy-Preserving Federated Learning Framework for Blockchain Networks. Clust. Comput..

[9] Phong L.T., Aono Y., Hayashi T., Wang L., Moriai S. (2017). Privacy-preserving deep learning: Revisited and enhanced. Proceedings of the International Conference on Applications and Techniques in Information Security, Auckland, New Zealand, 6–7 July 2017.

[10] Ma X., Ma J., Li H., Jiang Q., Gao S. (2018). PDLM: Privacy-preserving deep learning model on cloud with multiple keys. IEEE Trans. Serv. Comput..

[11] Li P., Li J., Huang Z., Li T., Gao C.Z., Yiu S.M., Chen K. (2018). Privacy-preserving outsourced classification in cloud computing. Clust. Comput..

[12] Wibawa F., Catak F.O., Kuzlu M., Sarp S., Cali U. (2022). Homomorphic encryption and federated learning based privacy-preserving CNN training: COVID-19 detection use-case. Proceedings of the 2022 European Interdisciplinary Cybersecurity Conference, Barcelona, Spain, 15–16 June 2022.

[13] Wang B., Li H., Guo Y., Wang J., Zhang Y. (2023). PPFLHE: A privacy-preserving federated learning scheme with homomorphic encryption for healthcare data. Appl. Soft Comput..

[14] Zhang X., Fu A., Wang H., Zhou Q., Zhang C. (2020). A privacy-preserving and verifiable federated learning scheme. Proceedings of the ICC 2020—2020 IEEE International Conference on Communications (ICC) Dublin, Ireland, 7–11 June 2020.

[15] Ma J., Naas S.A., Sigg S., Lyu X. (2022). Privacy-preserving federated learning based on multi-key homomorphic encryption. Int. J. Intell. Syst..

[16] Zhang L., Xu J., Vijayakumar P., Shen S., Zhang J. (2023). Homomorphic encryption-based privacy-preserving federated learning in IoT-enabled healthcare system. IEEE Trans. Netw. Sci. Eng..

[17] Huang Z., Bi Y., Zhang K., Hu B., Su Z., Tai C., Luan X. (2025). PDSA-FL: A Poisoning-Defense Secure Aggregation in Federated Learning. IEEE Trans. Inf. Forensics Secur..

[18] Fu Y., Niu X., Zhou L., Cai X., Yu F.R., Cheng N., Li C. (2025). A Hierarchical Blockchain-Enabled Secure Aggregation Algorithm for Federated Learning in IoV. IEEE Internet Things J..

[19] Wang T., Jin Y., Yang Q., Xia Y., Shi L., Zhang S. (2025). Zero-Knowledge Federated Learning: A New Trustworthy and Privacy-Preserving Distributed Learning Paradigm. arXiv.

[20] Andriambelo N.H., Moradpoor N. (2025). Privacy-Preserving Knowledge Graph Sharing in Peer-to-Peer Decentralized Federated Learning for Connected Autonomous Vehicles. Proceedings of the International Conference on Availability, Reliability and Security, Ghent, Belgium, 11–14 August 2025.

[21] Sun H., Bai T., Li J., Zhang H. (2025). zkDL: Efficient Zero-Knowledge Proofs of Deep Learning Training. IEEE Trans. Inf. Forensics Secur..

[22] Wu S., Li K., Zhang C. (2024). FL-R: An Interoperability Solutions for Blockchain Federated Learning. Proceedings of the 2024 7th International Conference on Data Storage and Data Engineering, Shanghai China, 27–29 February 2024.

[23] Wang G. (2019). Interpret Federated Learning with Shapley Values. arXiv.

[24] An J., Tang S., Sun X., Gui X., He X., Wang F. (2024). FREB: Participant Selection in Federated Learning with Reputation Evaluation and Blockchain. IEEE Trans. Serv. Comput..

[25] Wang Y., Kantarci B., Mardini W. (2021). Aggregation of Incentivized Learning Models in Mobile Federated Learning Environments. IEEE Netw. Lett..

[26] Rehman M.H.U., Salah K., Damiani E., Svetinovic D. (2020). Towards Blockchain-Based Reputation-Aware Federated Learning. Proceedings of the Conjunction with IEEE Infocom, EdgeBlock 2020: International Symposium on Edge Computing Security and Blockchain, Toronto, ON, Canada, 6–9 July 2020.

[27] Rizk E., Vlaski S., Sayed A.H. (2020). Dynamic Federated Learning. Proceedings of the 2020 IEEE 21st International Workshop on Signal Processing Advances in Wireless Communications, Atlanta, GA, USA, 26–29 May 2020.

[28] Yazdinejad A., Mohammadabadi Z.D., Dehghantanha A., Srivastava G. (2025). An Explainable and Privacy-Preserving Federated Learning Model for Threat Detection in Cyber-Physical-Social Systems. IEEE Trans. Comput. Soc. Syst..

[29] Kang J., Xiong Z., Niyato D., Xie S., Zhang J. (2019). Incentive Mechanism for Reliable Federated Learning: A Joint Optimization Approach to Combining Reputation and Contract Theory. IEEE Internet Things J..

[30] Zhang C., Xie Y., Bai H., Yu B., Li W., Gao Y. (2021). A survey on federated learning. Knowledge-Based Syst..

[31] Li T., Sahu A.K., Talwalkar A., Smith V. (2020). Federated Learning: Challenges, Methods, and Future Directions. IEEE Signal Process. Mag..

[32] Jiang W., Cheng C., Zhong C., Yue Q. (2025). An incentive mechanism based on game theory in Optimistic Rollup. Procedia Comput. Sci..

[33] Sun K., Wu J., Li J. (2024). Reputation-Aware Incentive Mechanism of Federated Learning: A Mean Field Game Approach. Proceedings of the 2024 9th IEEE International Conference on Smart Cloud (SmartCloud), New York, NY, USA, 10–12 May 2024.

[34] Abdi G.H., Sheikhani A.H.R., Kordrostami S., Ghane A., Babaie S. (2024). A novel selfish node detection based on reputation and game theory in Internet of Things. Computing.

[35] Groth J. (2016). On the Size of Pairing-Based Non-interactive Arguments. Proceedings of the 35th Annual International Conference on the Theory and Applications of Cryptographic Techniques, Vienna, Austria, 8–12 May 2016.

[36] Xu Y., Tan Y., Zhang C., Sun P., Zhang Y., Ren J., Jiang H., Zhang Y. (2025). Towards Privacy-Enhanced and Robust Clustered Federated Learning. IEEE Trans. Mob. Comput..

